# Dicarbonyl-modified lipoproteins contribute to proteinuric kidney injury

**DOI:** 10.1172/jci.insight.161878

**Published:** 2022-11-08

**Authors:** Jianyong Zhong, Hai-Chun Yang, Elaine L. Shelton, Taiji Matsusaka, Amanda J. Clark, Valery Yermalitsky, Zahra Mashhadi, Linda S. May-Zhang, MacRae F. Linton, Agnes B. Fogo, Annet Kirabo, Sean S. Davies, Valentina Kon

**Affiliations:** 1 Department of Pediatrics and; 2 Department of Pathology, Microbiology and Immunology, Vanderbilt University Medical Center, Nashville, Tennessee, USA.; 3 Institute of Medical Sciences and Department of Molecular Life Sciences, Tokai University School of Medicine, Kanagawa, Japan.; 4 Department of Pharmacology, Division of Clinical Pharmacology,; 5 Department of Medicine, and; 6 Department of Molecular Physiology and Biophysics, Vanderbilt University Medical Center, Nashville, Tennessee, USA.

**Keywords:** Nephrology, Cholesterol, Chronic kidney disease, Lymph

## Abstract

Lipoprotein modification by reactive dicarbonyls, including isolevuglandin (IsoLG), produces dysfunctional particles. Kidneys participate in lipoprotein metabolism, including tubular uptake. However, the process beyond the proximal tubule is unclear, as is the effect of kidney injury on this pathway. We found that patients and animals with proteinuric injury have increased urinary apolipoprotein AI (apoAI), IsoLG, and IsoLG adduct enrichment of the urinary apoAI fraction compared with other proteins. Proteinuric mice, induced by podocyte-specific injury, showed more tubular absorption of IsoLG-apoAI and increased expression of lipoprotein transporters in proximal tubular cells compared with uninjured animals. Renal lymph reflects composition of the interstitial compartment and showed increased apoAI and IsoLG in proteinuric animals, supporting a tubular cell-interstitium-lymph pathway for renal handling of lipoproteins. IsoLG-modified apoAI was not only a marker of renal injury but also directly damaged renal cells. IsoLG-apoAI increased inflammatory cytokines in cultured tubular epithelial cells (TECs), activated lymphatic endothelial cells (LECs), and caused greater contractility of renal lymphatic vessels than unmodified apoAI. In vivo, inhibition of IsoLG by a dicarbonyl scavenger reduced both albuminuria and urinary apoAI and decreased TEC and LEC injury, lymphangiogenesis, and interstitial fibrosis. Our results indicate that IsoLG-modified apoAI is, to our knowledge, a novel pathogenic mediator and therapeutic target in kidney disease.

## Graphical abstract

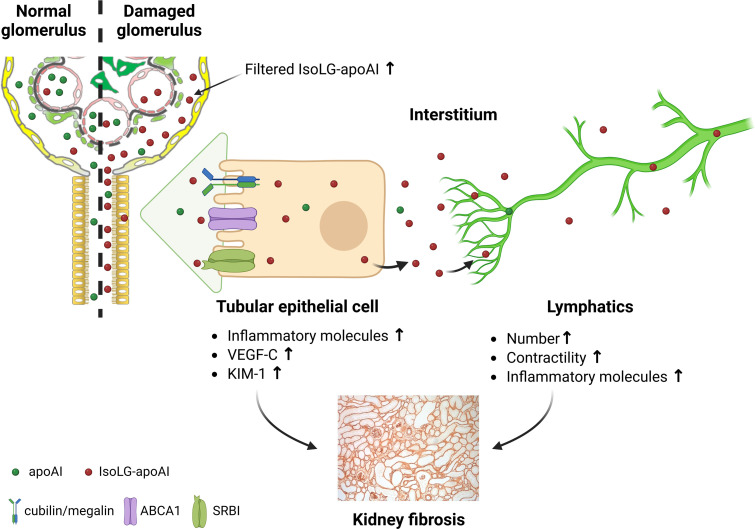

## Introduction

The beneficial effects of HDL have been extensively studied in cells involved in atherosclerosis, such as macrophages and endothelial cells. Recent emphasis has shifted from the quantity of HDL to the functionality of the HDL particles, which is affected by structural and chemical changes transforming the lipoproteins into harmful molecules (1–9). A key mechanism in the transformation involves reactive carbonyls formed from peroxidation of arachidonic acid that selectively form adducts with primary amines such as lysyl residues of proteins. Members of this family include malondialdehyde (MDA), 4-hydroxynonenal (4-HNE), 4-oxo-neonenal (4-ONE), and isolevuglandin (IsoLG). HDL modified by these reactive dicarbonyls causes toxicity that impairs the antiatherogenic and the beneficial extracardiac properties of HDL (10–13). Kidney disease alters the composition and functionality of HDL thought to contribute to increased atherosclerotic cardiovascular risk but also to progressive kidney damage (14).

Because of their hydrophobic nature, lipids associate with proteins, most often albumin, which in the kidney disease setting can lead to lipid deposition throughout the renal parenchyma. While albuminuria has long been viewed as a mechanism of kidney damage, accumulating evidence indicates it is not albumin itself, but rather the fatty acid cargo that is the critical factor driving albuminuric cytotoxicity (15–17). Little is known about other lipids and proteins that may be involved in kidney damage. Recently, we found that albuminuric children with various types of kidney injuries have increased urinary apolipoprotein AI (apoAI), the most abundant protein in the HDL particle, and biopsy samples showed prominent expression of apoAI in the proximal tubules (18). These findings are consistent with the understanding that proximal tubules reabsorb apoAI and HDL (19); however, what is currently unknown is the disposition of lipoproteins beyond the proximal tubule. This is an important biological concern since lipoprotein clearance from the interstitium throughout the body occurs primarily through the lymphatic network, which is increasingly recognized to be a significant modulator in development and progression of many diseases (12, 20–22). Whether this occurs in the kidney is unknown. Also unknown is whether disease-modified lipoproteins modulate lymphatic vessel dynamics. Finally, although kidney disease is a high-lipid peroxidation condition that disrupts the circulating levels and composition of HDL (13), the role of reactive carbonyls in injury-induced lymphatic vessel response has not been examined. Our data identify apoAI modified by reactive dicarbonyls as a potentially novel mechanism of kidney damage. This injury involves stimulation of inflammatory factors by the proximal tubule, oxidative stress in lymphatic endothelial cells (LECs), and impaired contractility of lymphatic vessels that can promote stagnation of harmful molecules and cells in the kidney interstitium.

## Results

### Patients and animals with kidney injury have increased urinary apoAI and IsoLG.

**Figure 1:**
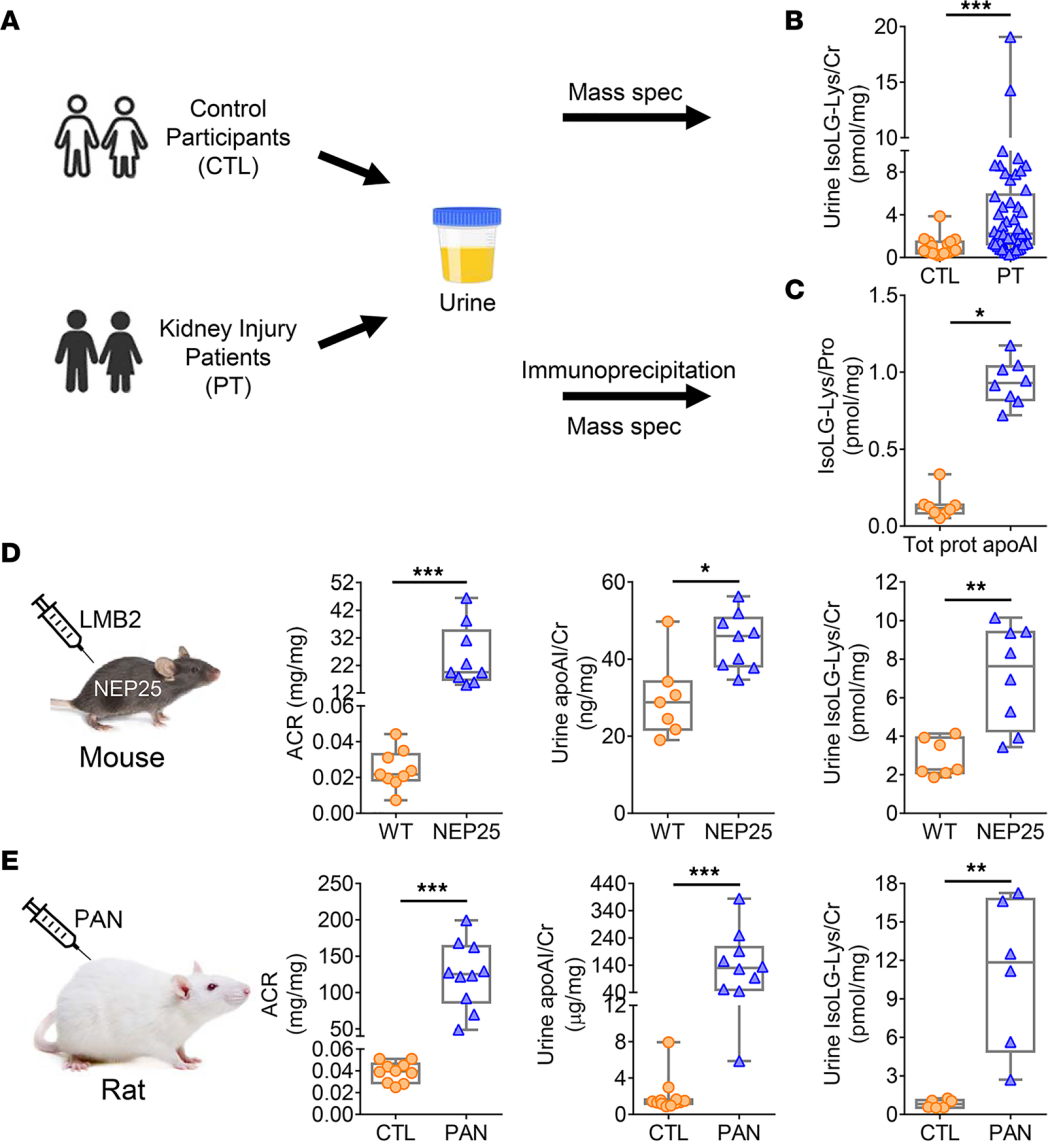
Kidney injury patients and animals have increased urinary apoAI and IsoLG. (**A**) The IsoLG adducts in urine and the IP apoAI fraction of patients (PTs) and matched CTLs were measured by mass spectrometry. (**B**) Compared with CTLs (*n* = 19), PTs (*n* = 50) had higher urinary IsoLG. (**C**) Urinary apoAI contained more IsoLG adducts versus total urinary proteins (*n* = 8). (**D**) Transgenic mice (NEP25) with albuminuria (ACR) following injection of toxin (LMB2) had significantly increased urinary apoAI and IsoLG versus WT mice. (**E**) Albuminuric puromycin-injected rats (PAN) had higher urinary apoAI and IsoLG versus CTL. *n* = 6–12 mice or rats/group. Data represented with box-and-whisker plot. Box plots show the interquartile range (box), median (line), and minimum and maximum (whiskers). Statistical significance determined by Wilcoxon rank sum test. *P < 0.05, **P < 0.01, ***P < 0.001.

Kidney injury increases oxidant stress and generation of lipid aldehydes, e.g., IsoLG. ApoAI is particularly susceptible to covalent modification by this aldehyde (23). Therefore, to determine if kidney injury affects urinary IsoLG in humans, we examined urine samples from our previously characterized cohort of patients with a spectrum of kidney injuries and high urinary excretion of apoAI and compared them with healthy control (CTL) participants (Figure 1A) (18). The clinical demographics for the participants have been reported and are shown in Supplemental Table 1 (supplemental material available online with this article; https://doi.org/10.1172/jci.insight.161878DS1). Subjects with elevated levels of urinary apoAI have higher urinary IsoLG-Lys than matched CTLs, assessed by mass spectrometry (Figure 1B). IP experiments confirmed that the apoAI fraction was markedly enriched with IsoLG adducts compared with the fraction containing all other urinary proteins (Figure 1C).

To determine how kidney injury affects renal handling of apoAI, we investigated several models of kidney injury in mice and rats. First, we studied transgenic mice that express human CD25 selectively in podocytes (NEP25). These mice develop primary podocyte injury and progressive albuminuria upon injection of anti-Tac (Fv)-PE38 (LMB2), an immunotoxin with specific binding to human CD25 (24). After injury was induced in NEP25 mice, confirmed by increased albuminuria (albumin/creatinine ratio [ACR]), urinary apoAI was increased compared with normal WT mice (Figure 1D). Paralleling the results in patients, urinary levels of IsoLG adducts were increased in albuminuric NEP25 mice compared with WT mice (Figure 1D). We also studied the puromycin aminonucleoside nephropathy (PAN) model, a well-known rat model of minimal change disease and focal and segmental glomerulosclerosis in humans (25). Albuminuric PAN rats had higher urinary apoAI and significant elevation in urinary IsoLG-Lys versus CTL rats (Figure 1E).

**Figure 2:**
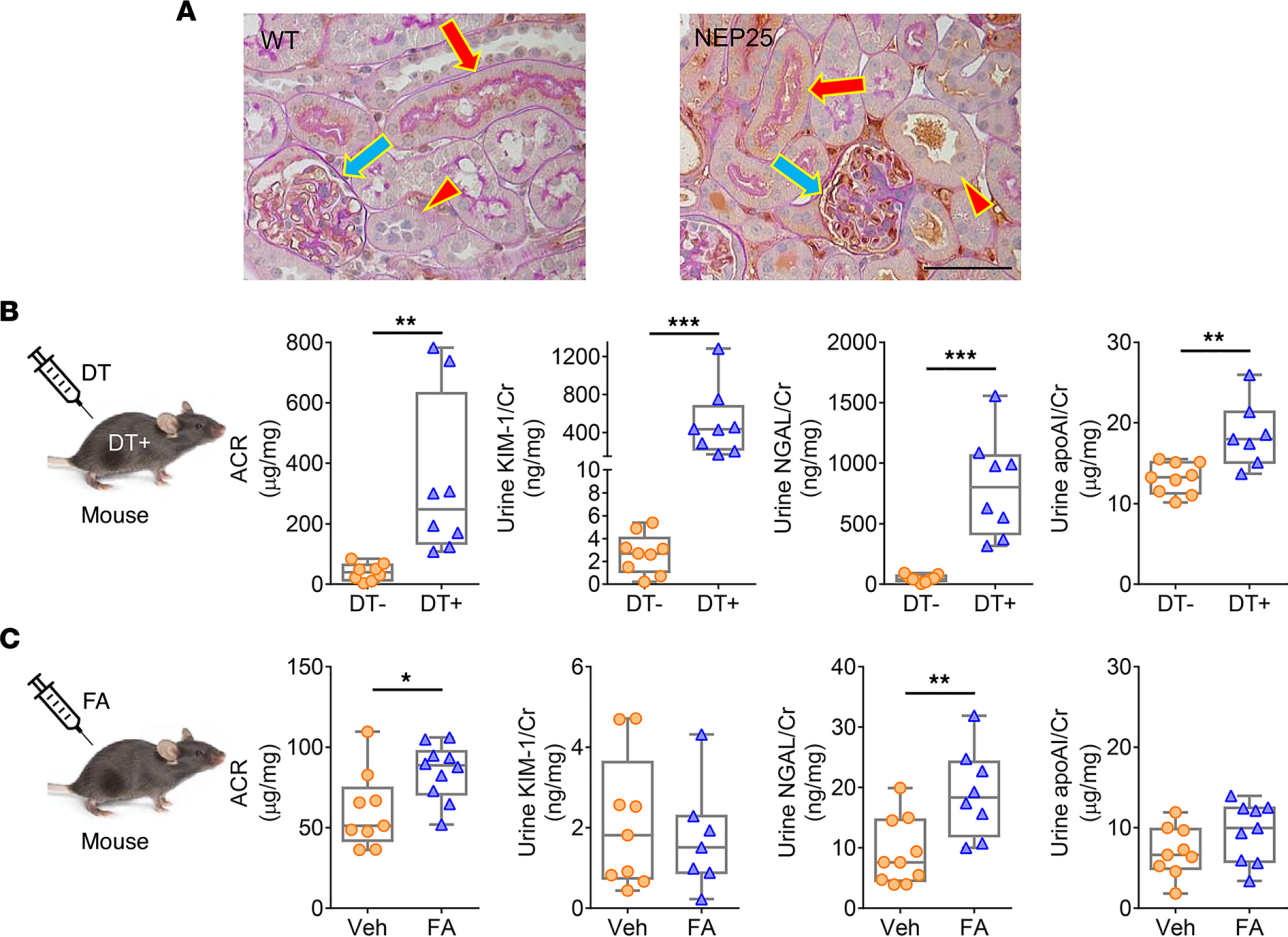
Distinct tubular handling of apoAI. (**A**) Proteinuric NEP25 kidneys showed greater apoAI within the glomerular capillary tuft (blue arrow) versus WT. ApoAI costaining with periodic acid–Schiff showed greater apoAI expression in proximal tubular cells (red arrow) than distal tubular cells (red arrowhead). Scale bar: 50 μm. (**B**) Proximal tubular injury following injection of DT^+^ increased ACR, urinary KIM-1, NGAL, and urinary apoAI versus DT^–^ mice. (**C**) FA-injected distal tubular injury mice had increased ACR and NGAL, but not KIM-1, and no change in urinary apoAI excretion. *n* = 6–12 mice/group. Data represent box-and-whisker plot. Box plots show the interquartile range (box), median (line), and minimum and maximum (whiskers). Statistical significance determined by Wilcoxon rank sum test. *P < 0.05, **P < 0.01, ***P < 0.001.

Kidneys from albuminuria NEP25 mice showed greater apoAI within the glomerular capillary tuft versus WT mice (Figure 2A). Further, apoAI expression was more prominent in proximal tubules (containing brush border) than in distal tubular cells (Figure 2A). To examine renal lipoprotein handling, we also studied tubular injury models without glomerular injury. Diphtheria toxin^+^ (DT^+^) transgenic mice express the DT receptor (DTR) only on proximal TECs (26) and develop acute kidney injury with proximal tubular injury following DT injection with increased ACR, urinary kidney injury molecule-1 (KIM-1), and neutrophil gelatinase-associated lipocalin (NGAL) (Figure 2B). These DT^+^ mice showed significantly higher urinary apoAI versus DT^–^ after DT injection (Figure 2B). Urinary IsoLG of DT^+^ mice was not significantly increased compared with DT^–^ mice (6.72 ± 1.12 vs. 5.12 ± 0.70 pmol/creatinine, *P* NS), indicating urinary IsoLG is derived primarily from the glomerular filtrate. In contrast, the folic acid (FA) model of distal tubular injury evidenced by increased NGAL, but not increased KIM-1, showed no change in urinary apoAI (Figure 2C). The critical features of human and animal models are shown in Supplemental Table 2. These results suggest that kidney injury including glomerular and proximal, but not distal, tubular injury results in increased urinary apoAI and associated IsoLG.

### Renal accumulation of IsoLG-apoAI reflects avid tubular uptake and compromised removal by renal lymphatics.

**Figure 3:**
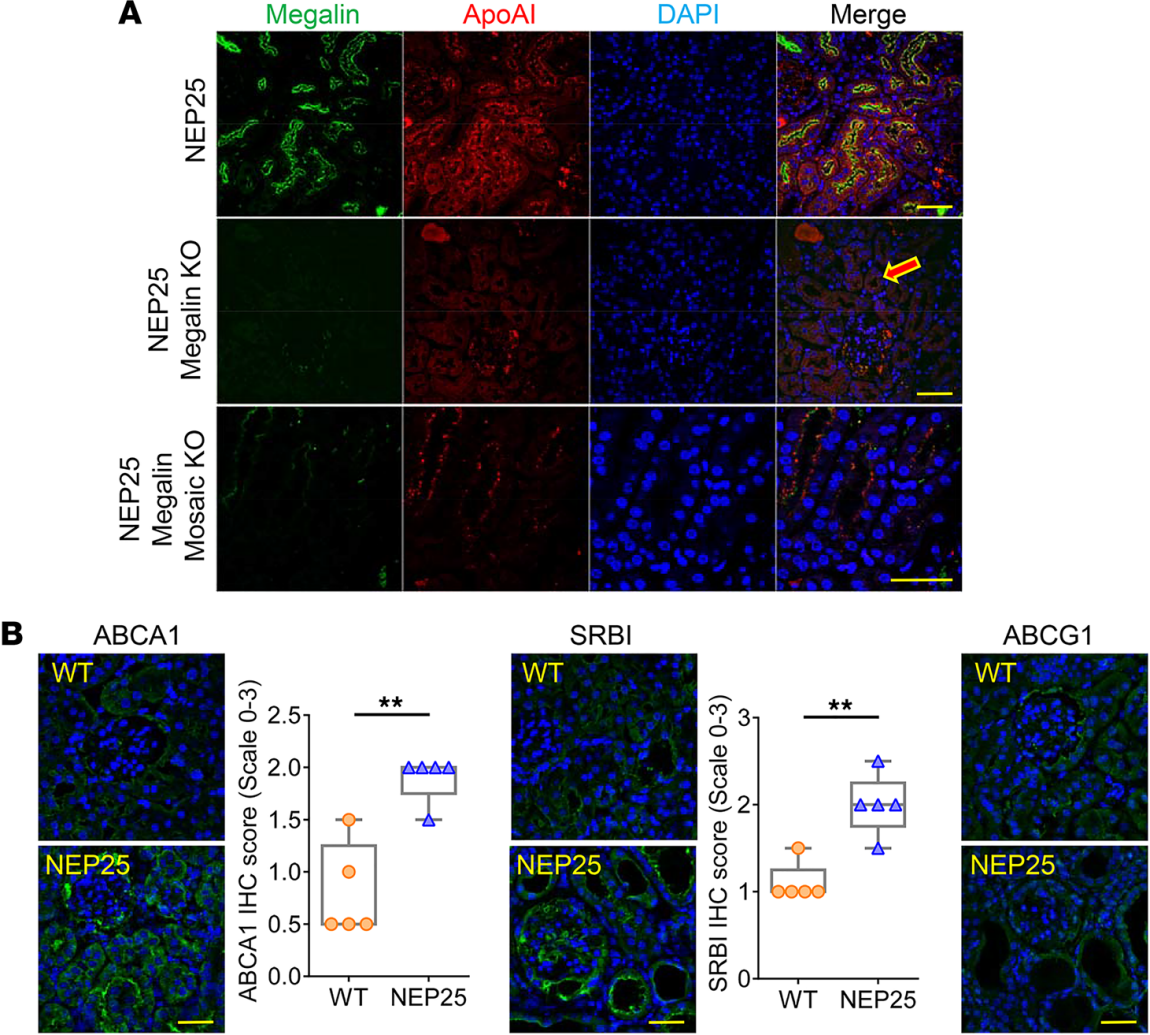
Pathways for tubular handling of apoAI. (**A**) Proximal tubular cells (megalin-positive, green) of NEP25 proteinuric kidneys absorbed apoAI (red, top panel). Uptake of apoAI (red) also occurred independently of megalin, illustrated in NEP25/megalin KO (middle panel) and NEP25/megalin mosaic KO mice (bottom panel). (**B**) IHC staining showed greater expression of lipoprotein transporters ABCA1 and SRBI, but not ABCG1, in NEP25 mice versus WT. Scale bar: 50 μm.

We next assessed tubular handling of apoAI and IsoLG-apoAI. ApoAI colocalized with megalin-positive cells (Figure 3A), corroborating megalin’s established role in apoAI reabsorption. However, while megalin is key in tubular uptake of apoAI, NEP25/megalin KO mice also showed apoAI in the proximal tubules (Figure 3A). Furthermore, NEP25/megalin mosaic KO mice exhibited apoAI in the megalin-deficient portions of proximal tubules (Figure 3A) (27). These findings suggest a megalin-independent pathway for apoAI reabsorption. Examination of the pathways involved in apoAI transport is illustrated in Figure 3B. Quantitation showed increased expression of ATP-binding cassette subfamily A member 1 (ABCA1) and scavenger receptor class B member 1 (SRBI) in tubules of proteinuric NEP25 mice versus WT mice. ATP-binding cassette subfamily G member 1 (ABCG1) expression was subtle and appeared unaffected by the proteinuric injury (Figure 3B).

**Figure 4:**
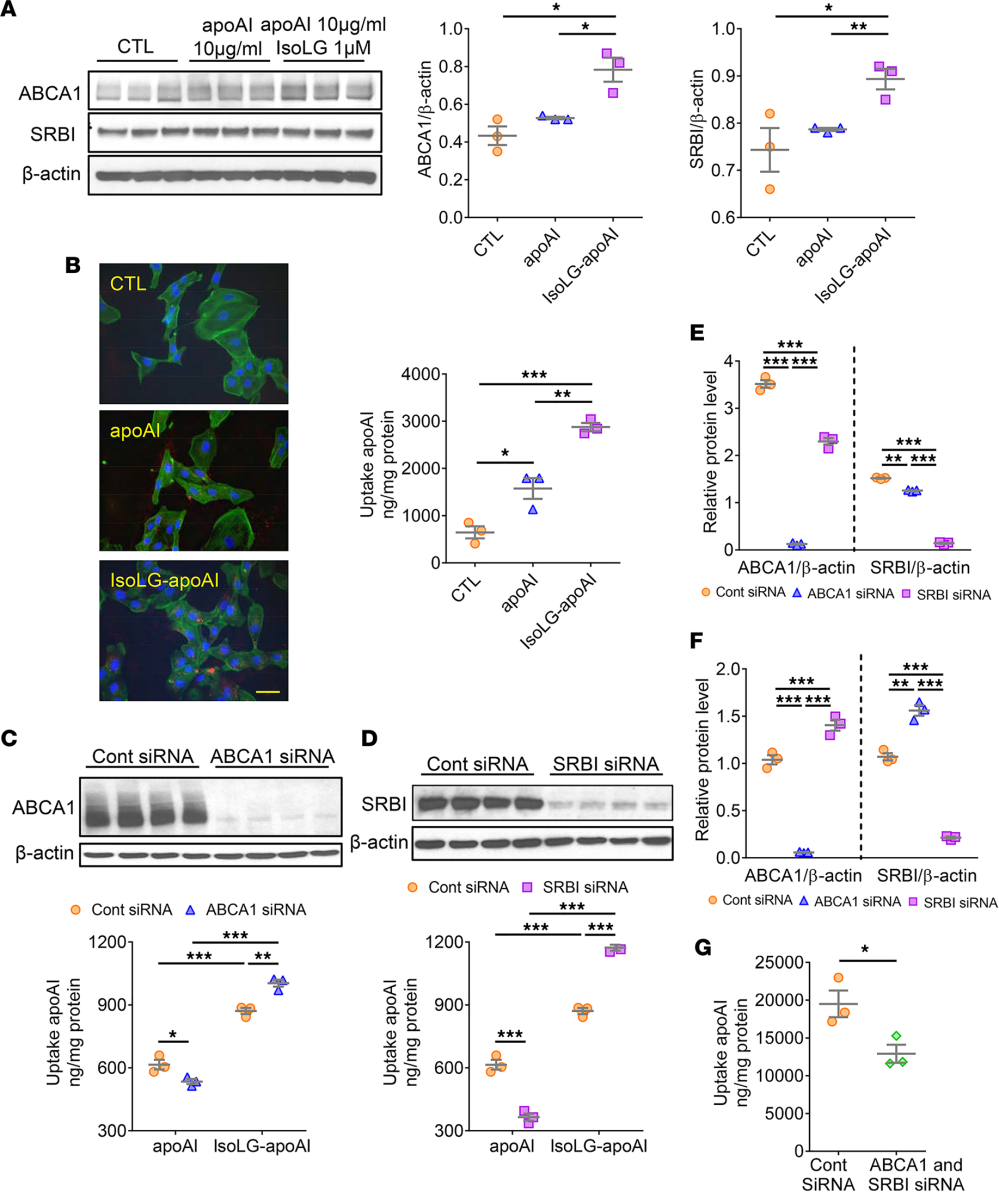
Distinct tubular uptake of apoAI versus IsoLG-apoAI. (**A**) Cultured TECs exposed to IsoLG-apoAI showed higher expression of ABCA1 and SRBI versus apoAI. (**B**) TECs took up more IsoLG-apoAI versus apoAI. Scale bar: 50 μm. Knockdown of either ABCA1 or SRBI (**C** and **D**) reduced TEC uptake of apoAI but not IsoLG-apoAI. (**E**) In TECs exposed to apoAI, knockdown of ABCA1 or SRBI decreased expression of the other transporter. (**F**) In TECs exposed to IsoLG-apoAI, ABCA1 knockdown significantly increased SRBI expression. SRBI siRNA significantly increased ABCA1 expression. (**G**) Knockdown of both ABCA1 and SRBI reduced cellular uptake IsoLG-apoAI. In vitro, experiments were performed independently 3 times with 3 wells per treatment. Data represent mean ± SEM. Overall statistical difference determined by Kruskal-Wallis test and pairwise difference by Wilcoxon rank sum test followed by Bonferroni correction on *P* values. *P < 0.05, **P < 0.01, ***P < 0.001.

Cultured human renal proximal tubule cells (TECs) with low megalin expression, exposed to IsoLG-apoAI versus apoAI, showed upregulated expression of ABCA1 and SRBI mRNA (ABCA1: 1.98 ± 0.13 vs. 1.21 ± 0.13; and SRBI: 1.44 ± 0.09 vs. 1.12 ± 0.10, both, *P* < 0.05). In parallel, both ABCA1 and SRBI proteins were significantly elevated by IsoLG-apoAI versus apoAI (Figure 4A). Functional assessment revealed that TECs accumulated more IsoLG-apoAI than apoAI (Figure 4B). To confirm ABCA1 and SRBI contribution to apoAI reabsorption, we used siRNA to knock down TEC expression of ABCA1 and SRBI. Knockdown of ABCA1 and SRBI was successful, with a greater than 80% reduction in ABCA1 and SRBI protein levels. Knockdown of ABCA1 or SRBI reduced TEC uptake of apoAI but not IsoLG-apoAI (Figure 4, C and D). To examine the possibility of a compensatory response, we knocked down 1 pathway and checked the effects on the other pathway. Unlike apoAI, IsoLG-apoAI increased SRBI expression after ABCA1 KD. Similarly, IsoLG-apoAI caused increased ABCA1 expression after SRBI KD (Figure 4, E and F). Knockdown of both ABCA1 and SRBI reduced TEC uptake of IsoLG-apoAI (Figure 4G). Together, these in vivo and in vitro studies suggest that proteinuric injury upregulates tubular expression of ABCA1 and SRBI, which augments reabsorption of filtered IsoLG-apoAI versus apoAI.

**Figure 5:**
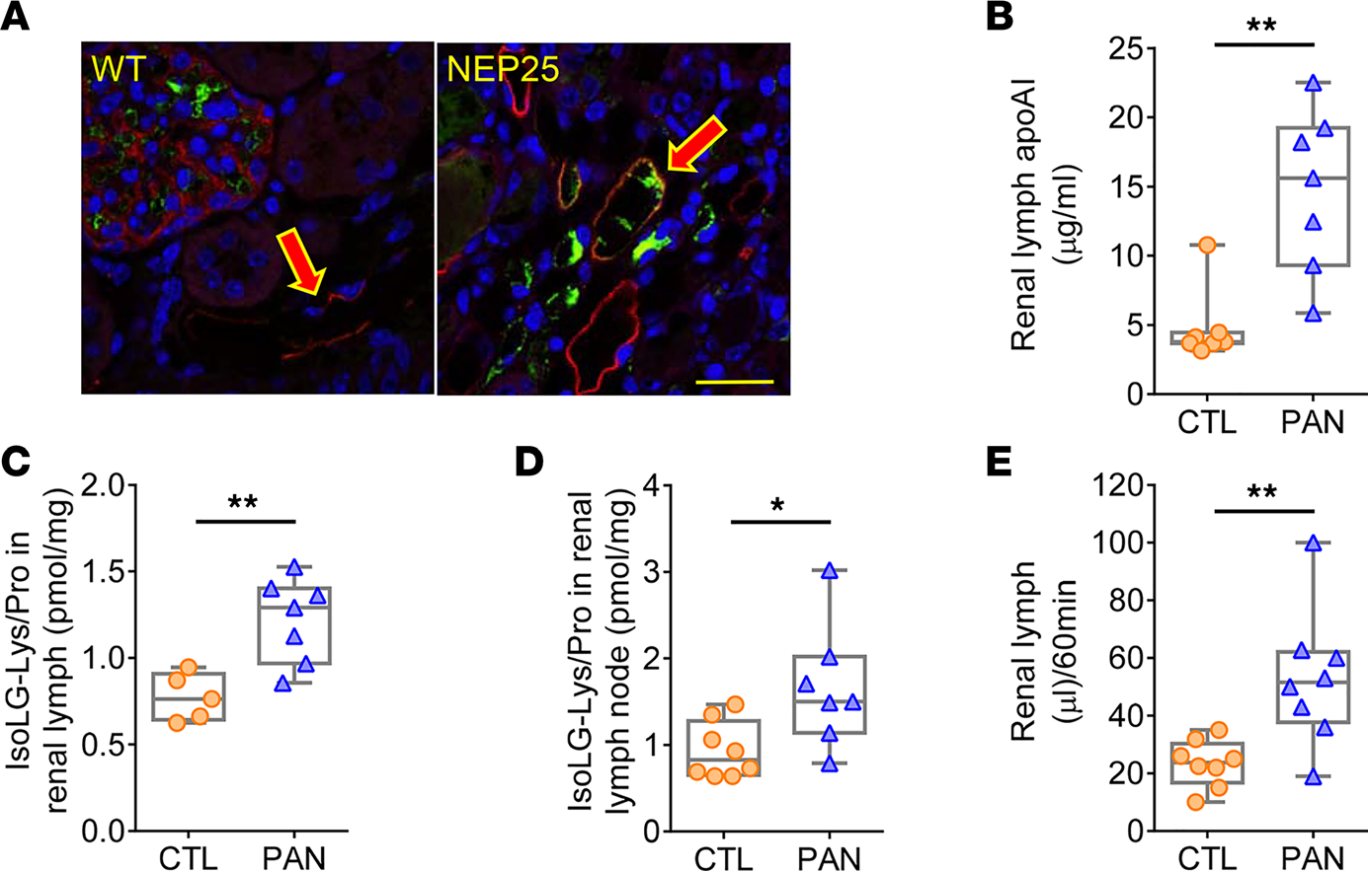
Renal transport of apoAI and IsoLG-apoAI involves lymphatic vessels. (**A**) ApoAI (green) immunostaining was increased in podoplanin-positive (LEC marker, red) lymphatic vessels of proteinuric NEP25 versus WT mice. Scale bar: 50 μm. (**B**) Renal lymph of proteinuric PAN rats had increased apoAI versus CTL. (**C** and **D**) Proteinuric PAN rats had increased IsoLG adducts in renal lymph and renal lymph nodes versus CTL. (**E**) Renal lymphatic flow in PAN was higher versus CTL rats. *n* = 5–8 rats/group. Data represent box-and-whisker plot. Box plots show the interquartile range (box), median (line), and minimum and maximum (whiskers). Statistical significance determined by Wilcoxon rank sum test. * *P* < 0.05, **P < 0.01.

Since lymphatic vessels are the primary pathway for transporting lipoproteins from the peripheral interstitial space, we next examined the role of renal lymphatics in conveying lipoproteins taken up by tubules into the renal interstitium (28). ApoAI localized in podoplanin-positive lymphatic vessels, which were more prominent in NEP25 mice compared with WT mice (Figure 5A). To directly ascertain the composition of renal lymph, we used our rat proteinuric injury model where lymph collection is more accessible. Renal lymph of PAN rats had higher levels of apoAI versus CTL (Figure 5B), complementing the conspicuous colocalization of apoAI within renal lymphatic vessels observed in NEP25 mice (Figure 5A). IsoLG levels were higher in renal lymph of PAN rats (Figure 5C). The lymph nodes downstream from the kidney in PAN rats contained higher IsoLG versus CTL animals (Figure 5D). Next, we compared the renal lymphatic vessel function in PAN versus CTL rats. PAN rats had increased renal lymph flow (Figure 5E).

**Figure 6:**
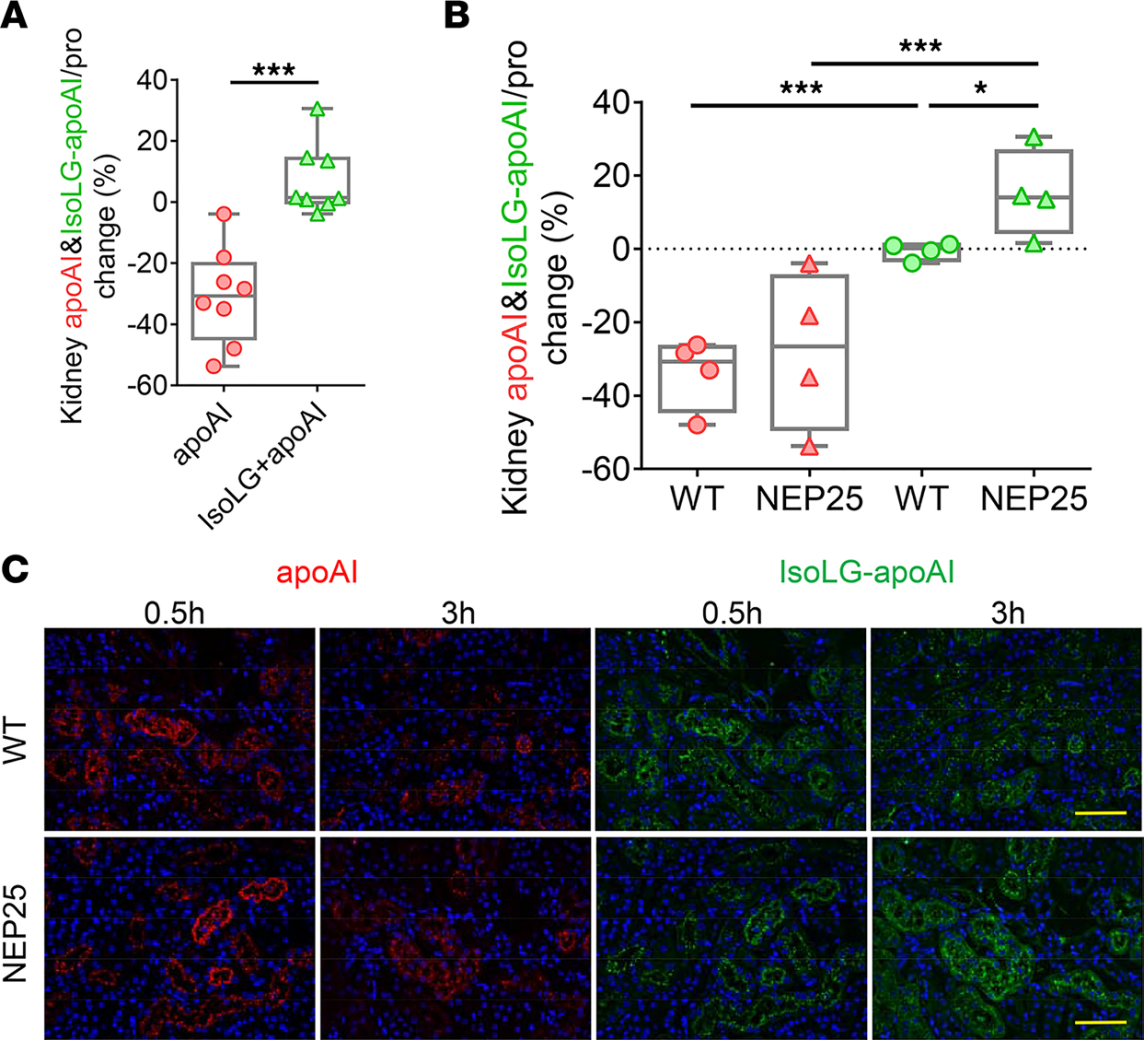
Proteinuric NEP25 mice show accumulation of IsoLG-apoAI versus apoAI. (**A**) Kidney clearance of fluorescence-labeled apoAI (red) was more rapid compared with IsoLG-apoAI (green). (**B**) Fluorescence-labeled apoAI (red) was similarly cleared by NEP25 and WT mice. In contrast, fluorescence-labeled IsoLG-apoAI (green) persisted in NEP25 kidneys versus WT 3 hours after injection. (**C**) Representative kidney images of fluorescent apoAI (red) and IsoLG-apoAI (green). Scale bar: 50 μm. *n* = 4 mice/group. Data represent box-and-whisker plot. Box plots show the interquartile range (box), median (line), and minimum and maximum (whiskers). Statistical significance determined by Kruskal-Wallis test and Wilcoxon rank sum test on *P* values. * *P* < 0.05, ***P < 0.001.

To determine how normal versus injured kidneys handle IsoLG-modified apoAI versus apoAI, we injected fluorescent apoAI (labeled with red dye) and IsoLG-apoAI (labeled with green dye) into WT and proteinuric NEP25 mice. In all kidneys, the renal apoAI change between 30 and 180 minutes was greater than the change in IsoLG-apoAI, indicating more rapid clearance of apoAI versus IsoLG-apoAI (Figure 6A). Comparison of WT and proteinuric kidneys revealed similar clearance of apoAI. In contrast, kidney clearance of IsoLG-apoAI was delayed in both WT and NEP25 kidneys versus apoAI (Figure 6B). The clearance of IsoLG-apoAI was especially delayed in NEP25 kidneys, which showed accumulated IsoLG-apoAI 3 hours after injection (Figure 6, B and C). These results demonstrate that IsoLG-apoAI is cleared more slowly than unmodified apoAI and that the delay in renal clearance is accentuated in proteinuric kidneys.

### Differential effects of apoAI versus IsoLG-apoAI on TECs, LECs, and lymphatic vessels.

**Figure 7:**
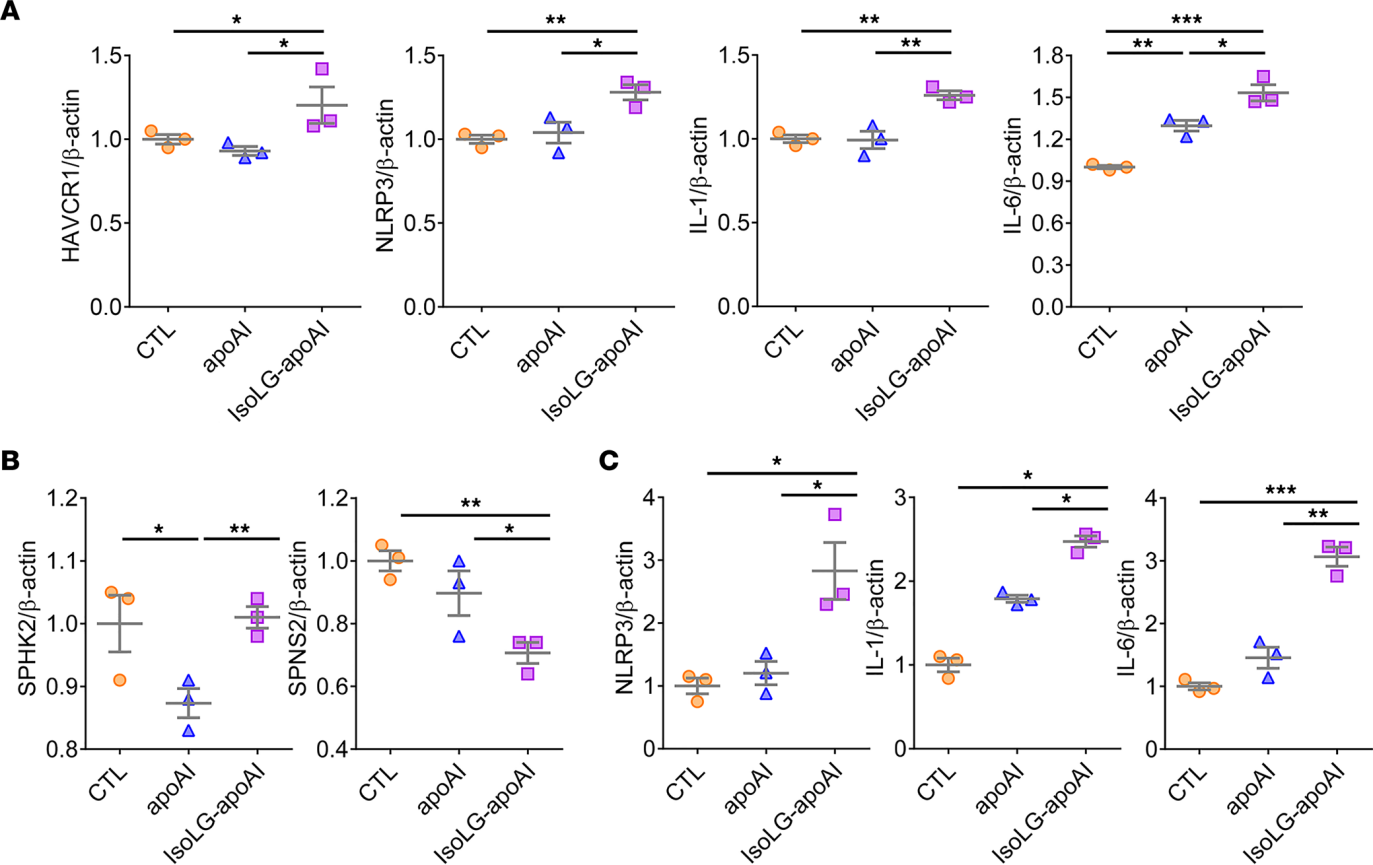
ApoAI and IsoLG-apoAI exert distinct effects on proximal TECs and LECs. (**A**) Cultured TECs exposed to IsoLG-apoAI increased KIM-1 (HAVCR1), NLRP3, IL-1, and IL-6 gene expression versus unmodified apoAI. (**B**) Cultured LECs exposed to IsoLG-apoAI increased SPHK2 mRNA and reduced SPNS2 mRNA versus apoAI. (**C**) LECs exposed to IsoLG-apoAI increased NLRP3, IL-1, and IL-6 gene expression versus apoAI. In vitro, experiments were performed independently 3 times with 3 wells per treatment. Data represent mean ± SEM. For more than 2 groups, statistical significance was determined by Kruskal-Wallis test followed by Wilcoxon rank sum tests and Bonferroni correction on *P* values. *P < 0.05, **P < 0.01, ***P < 0.001.

To determine whether apoAI/IsoLG-apoAI is a contributor to kidney injury, TECs were exposed to apoAI or IsoLG-apoAI. IsoLG-apoAI increased TEC gene expression of KIM-1 (*HAVCR1*), a marker of tubular injury, compared with apoAI (Figure 7A). IsoLG-apoAI also increased markers of inflammation, including NLR family pyrin domain containing 3 (*NLRP3*), *IL-1*, and *IL-6* gene expression (Figure 7A). Cultured LECs exposed to IsoLG-apoAI showed higher sphingosine kinase 2 (*SPHK2*) and decreased sphingolipid transporter 2 (*SPNS2*) expression (Figure 7B), 2 key regulators of sphingosine-1-phosphate (S1P) production, which in turn stimulates proinflammatory cytokines (29). S1P levels in LECs and cellular supernatant were also elevated by IsoLG-apoAI versus apoAI (cells: 55.84 ± 8.90 vs. 32.74 ± 3.11; and supernatant: 146.35 ± 25.01 vs. 64.40 ± 1.18 ng/mg, respectively, both *P* < 0.05). LECs expressed more inflammatory factor genes, *NLRP3*, *IL-1*, and *IL-6*, when exposed to IsoLG-apoAI versus apoAI (Figure 7C). Thus, absorption of IsoLG-apoAI by epithelial cells promoted TEC/LEC injury and inflammation.

**Figure 8:**
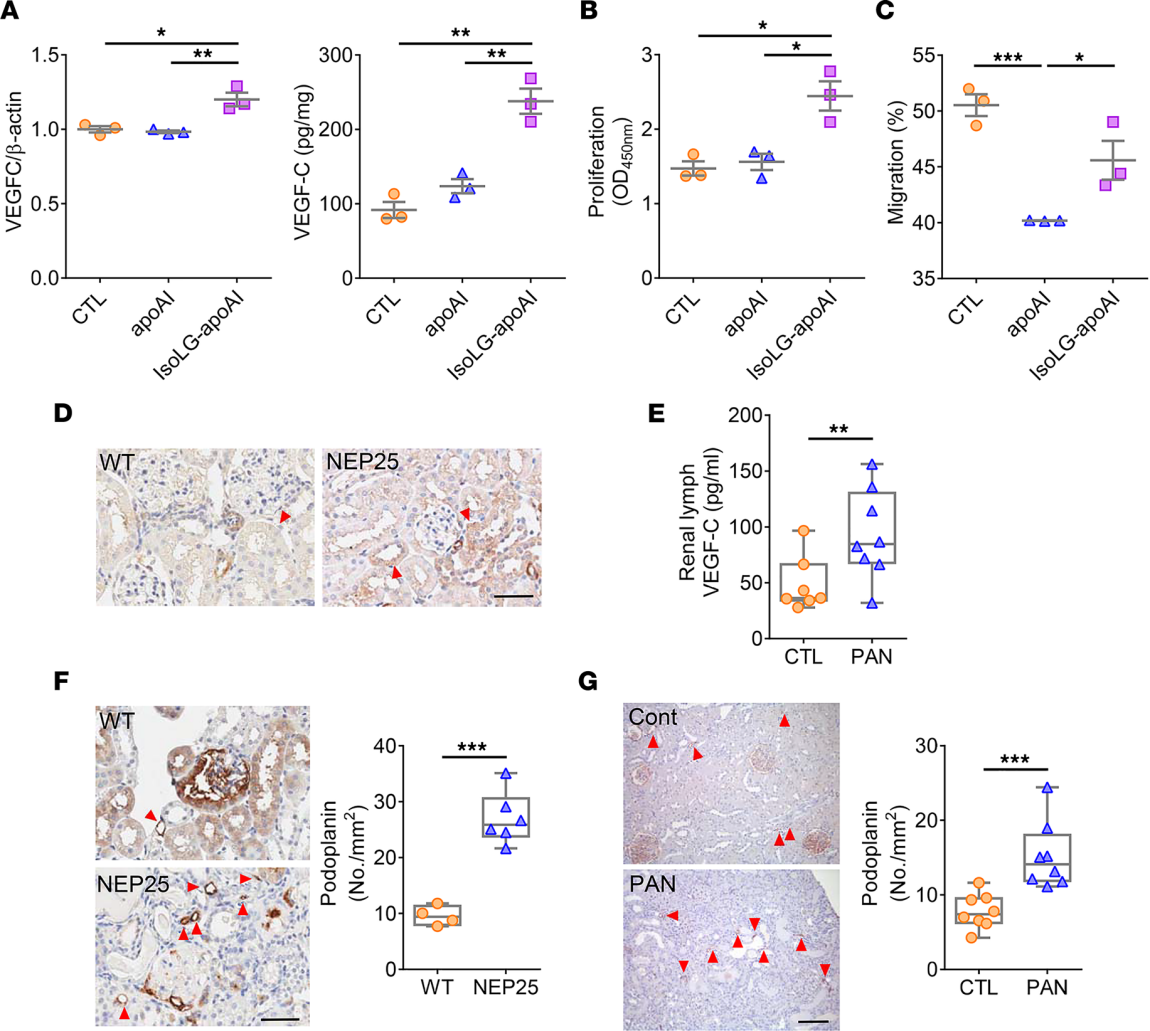
Distinct effects of apoAI versus IsoLG-apoAI on lymphangiogenesis. (**A**) Cultured TECs exposed to IsoLG-apoAI expressed more VEGFC mRNA and produced more VEGF-C protein versus apoAI. (**B**) IsoLG-apoAI increased LECs proliferation versus apoAI. (**C**) IsoLG-apoAI increased LECs migration versus apoAI. (**D**) VEGF-C expression by immunostaining was increased in TECs in NEP25 versus WT mice. Scale bar: 50 μm. (**E**) VEGF-C in PAN renal lymph was higher versus CTL. (**F**) NEP25 kidneys showed increased interstitial podoplanin expression versus WT mice. Scale bar: 50 μm. (**G**) PAN injured rats showed increased interstitial podoplanin expression versus CTL. Scale bar: 100 μm. In vitro, experiments were performed independently 3 times with 3 wells per treatment. *n* = 4–8 mice or rats/group. Data represent mean ± SEM for in vitro study and box-and-whisker plot for in vivo study. Box plots show the interquartile range (box), median (line), and minimum and maximum (whiskers). For 2 independent groups, statistical significance determined by Wilcoxon rank sum test. For more than 2 groups, statistical significance determined by Kruskal-Wallis test followed by Wilcoxon rank sum tests and Bonferroni correction on *P* values. *P < 0.05, **P < 0.01, ***P < 0.001.

Renal TECs produce VEGF-C, a growth factor that promotes lymphangiogenesis (30). IsoLG-apoAI significantly increased expression of VEGF-C (*VEGFC*) gene and protein in cultured TECs (Figure 8A). In LECs, IsoLG-apoAI increased proliferation compared with apoAI (Figure 8B). While apoAI decreased LEC migration, IsoLG-apoAI restored this parameter (Figure 8C). Complementing these results, in vivo studies showed greater immunostaining of VEGF-C in TECs of NEP25 mice versus WT mice (Figure 8D). PAN proteinuric rats had increased VEGF-C in renal lymph versus CTL animals (Figure 8E). In line with greater tubular production in VEGF-C, the renal lymphatic network of injured animals expanded. Lymphangiogenesis, quantitated by podoplanin staining, was significantly increased in both NEP25 mice and PAN rats compared with normal animals (Figure 8, F and G). Another LEC marker, lymphatic vessel endothelial hyaluronan receptor 1 (LYVE-1), also increased (WT: 1.31 ± 0.25 vs. NEP25: 2.40 ± 0.37 no./mm^2^, *P* < 0.05). These results demonstrate that IsoLG-apoAI promotes lymphangiogenesis by directly stimulating LEC proliferation and indirectly by activation of TEC generation of VEGF-C.

**Figure 9:**
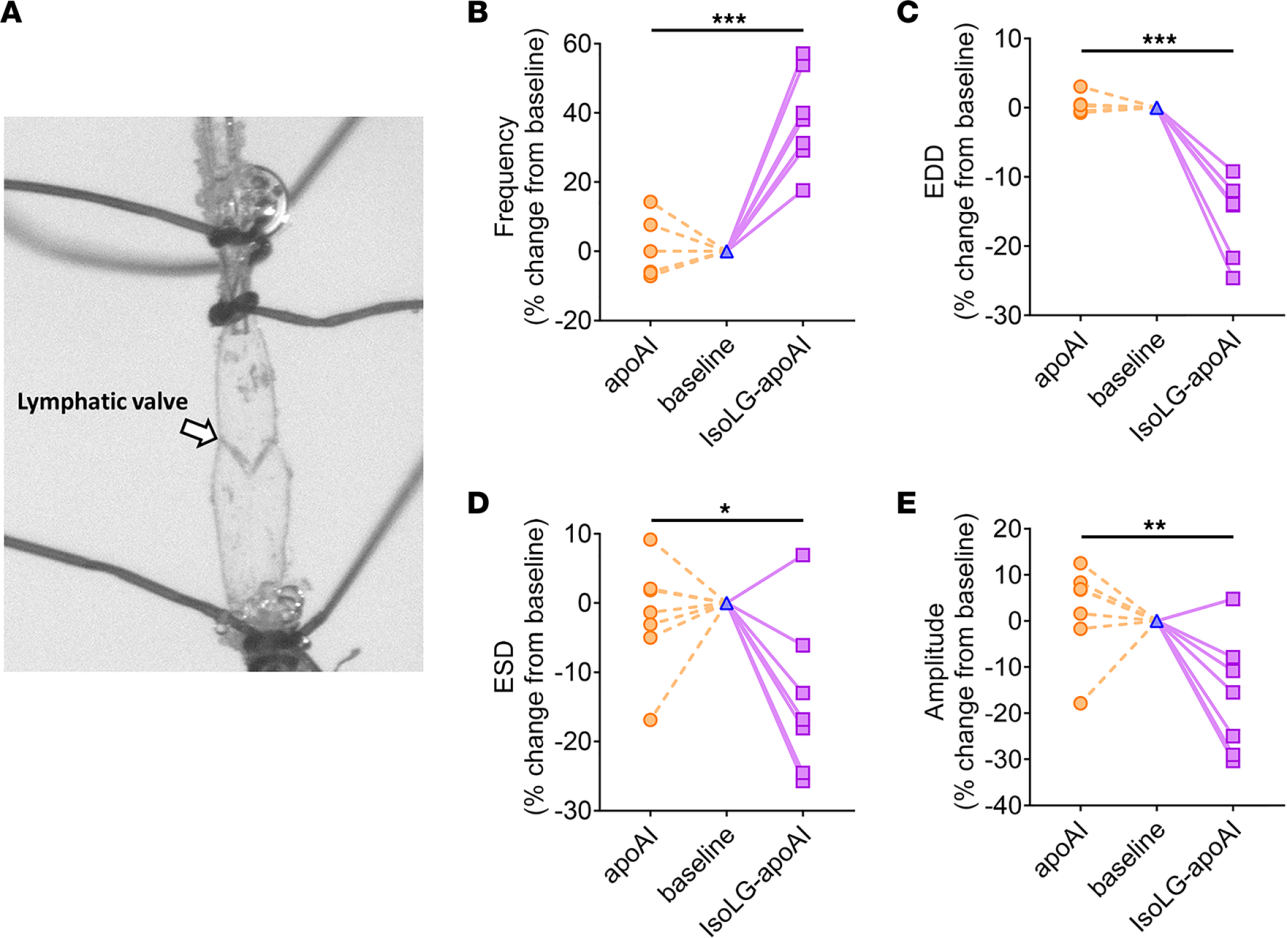
Distinct effects of apoAI versus IsoLG-apoAI on renal lymphatic vessel dynamics. (**A**) Renal lymphatic vessels were isolated, cannulated, and mounted in a perfusion chamber for pressure myography assays. Vasodynamic changes caused by unmodified apoAI (orange) are shown by the interrupted lines to the left of the baseline value, and changes caused by IsoLG-apoAI (purple) are shown by the solid lines to the right of the baseline value. Compared with the modest change with apoAI, IsoLG-apoAI caused a more prominent change in contraction frequency (**B**), EDD (**C**), ESD (**D**), and contraction amplitude (**E**). *n* = 8 rats/group. Statistical significance determined by Wilcoxon signed rank test. *P < 0.05, **P < 0.01, ***P < 0.001.

In ex vivo studies, isolated renal lymphatic vessels from rats showed that IsoLG-apoAI caused a more prominent change in contraction frequency, end-diastolic diameter (EDD), end-systolic diameter (ESD), and amplitude of contraction versus unmodified apoAI (Figure 9). Taken together, these results support the concept that proteinuric injury-driven renal accumulation of IsoLG-apoAI activates TECs, LECs, and lymphatic dynamics. The activated TECs and LECs produce more potentially harmful inflammatory cytokines and lymphangiogenic growth factors.

### Scavenging IsoLG lessens kidney injury.

**Figure 10:**
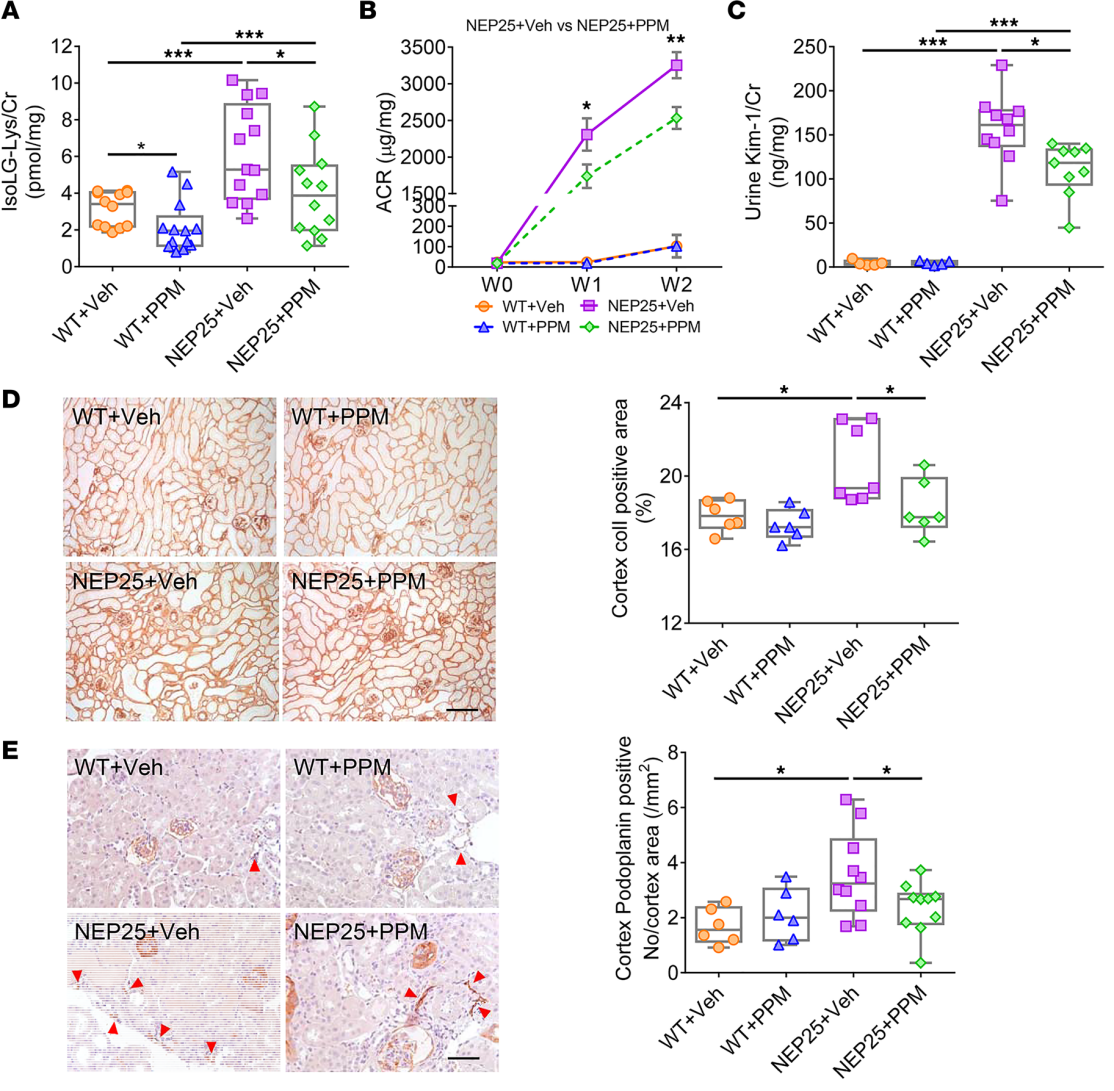
Dicarbonyl scavenger PPM improved kidney injury and attenuated lymphangiogenesis. PPM treatment decreased urinary IsoLG adducts (**A**), albuminuria (**B**), and KIM-1 (**C**) in NEP25 mice compared with NEP25 mice treated with vehicle. (**D**) PPM treatment in NEP25 mice decreased renal cortical collagen I (colI) expression compared with vehicle-treated NEP25 mice. Scale bar: 100 μm. (**E**) Podoplanin immunostaining showed PPM treatment reduced kidney lymphangiogenesis in NEP25 mice compared with vehicle-treated NEP25 mice. Scale bar: 50 μm. *n* = 3–13 mice/group. Data represent box-and-whisker plot. Box plots show the interquartile range (box), median (line), and minimum and maximum (whiskers). Statistical significance determined by Kruskal-Wallis test followed by Wilcoxon rank sum tests and Bonferroni correction on *P* values. *P < 0.05, **P < 0.01, ***P < 0.001.

Results in TECs and LECs indicate that the highly reactive lipid dicarbonyl IsoLG plays a role in renal injury. We therefore used the lipid dicarbonyl-reactive scavenger pentylpyridoxamine (PPM) to assess possible protective effects against proteinuric kidney injury. In vivo treatment of NEP25 mice with PPM significantly reduced urinary IsoLG (Figure 10A). Urinary F2-IsoP levels, a measure of systemic lipid peroxidation, were not affected by PPM (NEP25: 1.57 ± 0.07 vs. NEP25 + PPM: 1.79 ± 0.23 ng/mg, *P* NS). These results underscore that the PPM effects on kidney injury are not due to a general inhibition of peroxidation or chelation, but a specific effect of PPM to scavenge IsoLG. NEP25 mice treated with PPM also showed reduced albuminuria compared with vehicle-treated NEP25 mice (Figure 10B). Tubular injury, measured by KIM-1, was also lessened by PPM in NEP25 versus vehicle-treated NEP25 mice (Figure 10C). PPM significantly decreased interstitial fibrosis in kidney cortex (Figure 10D). The lymphatic network, quantitated by podoplanin and LYVE-1 staining (NEP25: 2.40 ± 0.37 vs. NEP25 + PPM: 1.05 ± 0.27 no./mm^2^, *P* < 0.01), was also reduced by PPM treatment in NEP25 mice versus vehicle-treated mice (Figure 10E).

**Figure 11:**
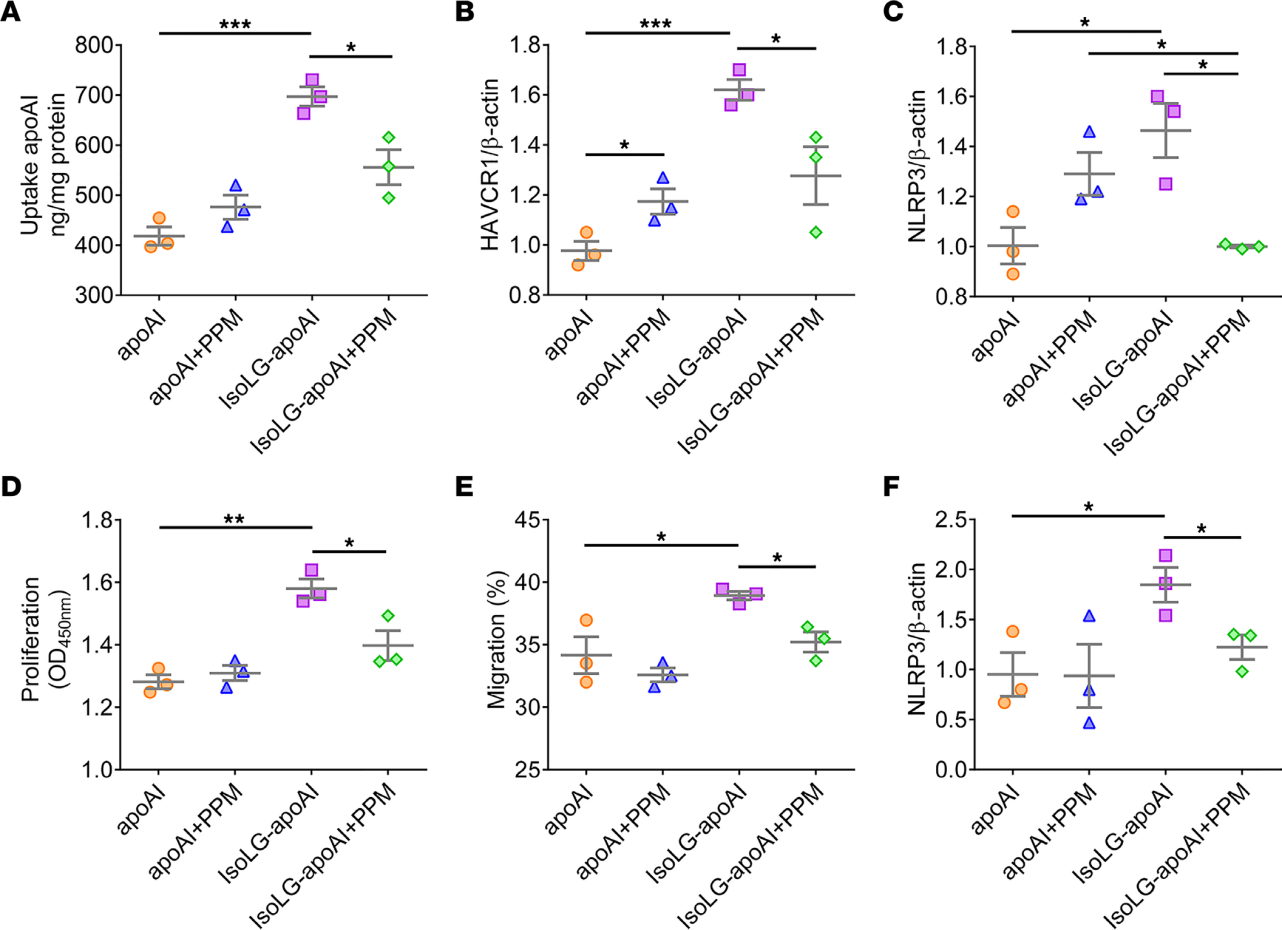
Dicarbonyl scavenger PPM lessened injury in proximal TECs and LECs. (**A**–**C**) In cultured TECs, PPM treatment reduced uptake of IsoLG-apoAI, KIM-1 (HAVCR1), and NLRP3 gene expression. (**D**–**F**) In cultured LECs, PPM treatment attenuated proliferation, migration, and NLRP3 gene expression. In vitro, experiments were performed independently 3 times with 3 wells per treatment. Data represent mean ± SEM. Statistical significance determined by Kruskal-Wallis test followed by Wilcoxon rank sum tests and Bonferroni correction on *P* values. *P < 0.05, **P < 0.01, ***P < 0.001.

In vitro studies further confirmed the protective effects of PPM on TECs and LECs. PPM treatment lessened TEC absorption of IsoLG-apoAI (Figure 11A) and decreased KIM-1 (*HAVCR1*) and *NLRP3* gene expression (Figure 11, B and C), suggesting reduction in the tubular epithelial cell injury and inflammation. In LECs exposed to IsoLG-apoAI, PPM also reduced proliferation (Figure 11D), migration (Figure 11E), and *NLRP3* gene expression (Figure 11F), suggesting a beneficial modulating effect of scavenging IsoLG on lymphatic cells.

## Discussion

Kidney disease leads to abnormalities in circulating lipids and lipoproteins through mechanisms involving disruption in lipid metabolism by the liver and impairment in maturational assembly in the plasma (13, 31). The kidneys themselves have a role in lipoprotein metabolism, although it remains uncertain if kidney disease disrupts the lipoprotein handling and if such disruptions have untoward kidney consequences. The current studies provide insights into kidney lipoprotein metabolism by showing that 1) experimental and human proteinuric kidney injuries show increased urinary apoAI enriched in the highly reactive dicarbonyl IsoLG; 2) kidney accumulation of IsoLG-apoAI in proteinuric disease reflects avid proximal tubule uptake and inadequate removal by the renal lymphatic vascular network; 3) IsoLG-apoAI not only damages TECs but also directly alters the phenotype of lymphatic vessel endothelial cells and disrupts the vasodynamic functions of kidney lymphatic vessels; and 4) scavenging IsoLG lessens tubular and LEC damage and reduces kidney injury.

We have previously shown that an important mechanism for degradation of lipoprotein quality involves IsoLG forming covalent adducts on lysines in apoAI/HDL, which compromises their antiatherogenic effects (11, 23). ApoAI/HDL also regulates inflammation, oxidation, proliferation, apoptosis, and vasodilation, processes that pertain to conditions beyond classic cardiovascular diseases, such as stroke, cardiac events, and atherosclerosis. These additional conditions include, e.g., sepsis, diabetes, obesity, rheumatoid arthritis, and cancer (13, 32). Kidney disease changes apoAI/HDL metabolism in ways that lead to lower levels and altered composition of apoAI/HDL that impairs the functionality of the particles (13). Whether modified apoAI/HDL gains access to the urinary space and whether this negatively affects the kidneys is unknown. Using samples from a previously reported cohort of albuminuric children with a spectrum of kidney disease and elevated urinary apoAI (18), we found higher IsoLG adducts compared with healthy CTLs. Critically, IP of the urinary apoAI fraction showed a 7-fold enrichment of IsoLG adducts in the apoAI fraction compared with IsoLG adducts in the fraction containing other urinary proteins (Figure 1C). This clinical observation prompted us to examine albuminuric animal models to understand how injuries along the nephron, including glomeruli, proximal, and distal tubules impact renal handling of IsoLG-apoAI. Our data show that albuminuric NEP25 mice and PAN rats have increased urinary apoAI associated with increased urinary IsoLG. IP of human urine samples showed that the apoAI fraction, but not the fraction containing other proteins, is the most enriched protein in IsoLG. Thus, in addition to the recognized effects of disease to increase IsoLG-modified apoAI/HDL in plasma, our results reveal that not only normal apoAI but also IsoLG-apoAI appear in urine of animals and humans with albuminuric injury. The results expand the concept of urinary lipoproteins as a marker/mechanism of progressive kidney damage that heretofore has focused on free fatty acids injury of proximal tubular cells (16, 33). Our data suggest that beyond free fatty acids associated with albumin, IsoLG-modified apoAI is a potentially novel candidate that can directly injure kidney parenchymal cells. To our knowledge, these effects have not been previously described and transcend the well-known adverse consequences of modified apoAI that include reduced cholesterol efflux, blunted antioxidant function, and augmented inflammatory response (10).

Although we find elevated IsoLG in urine and kidney, we did not specifically address the site(s) where IsoLG formed. Increased level of IsoLG adducts has been documented in plasma lipoproteins of patients with familial hypercholesterolemia, in circulating monocytes of hypertensive humans, and in murine atherosclerosis plaques (11, 34). Our recent study in PAN proteinuric rats (12) showed increased apoAI in intestinal lacteals, mesenteric lymph, and plasma, suggesting kidney damage stimulates apoAI production. This stimulation in extrarenal production of apoAI may relate to increased urinary loss of apoAI in proteinuric kidney injury. It is possible that, similar to albuminuria stimulating hepatic production of albumin, urinary apoAI losses stimulate production in the 2 organs responsible for its synthesis, namely liver and intestine. The current study focused on the kidney, underscoring IsoLG-apoAI that can be harmful to a variety of cells at various sites accumulating these molecules, including the kidneys.

The increased urinary apoAI and IsoLG-apoAI in proteinuric injury was coupled with distinct handling of the unmodified and modified apoAI along the nephron. First, the higher levels of urinary apoAI and IsoLG-apoAI in NEP25 mice and PAN rats were paralleled by greater abundance of apoAI in proximal tubules versus normal CTLs. A key role of the proximal tubules was further underscored by the findings that urinary apoAI was increased by proximal tubule injury in DT transgenic mice but not by distal tubular injury in the FA model. These results parallel our previous observations that children with glomerular disease and proximal tubule disorders, but not distal tubular disorders, have elevated urinary apoAI (18). In the proximal tubule, the primary process for reabsorption of apoAI from the glomerular filtrate is the megalin-cubilin pathway (35). Our results show that in proteinuric disease, filtered apoAI is also reabsorbed via megalin-independent pathways involving ABCA1/SRBI, which preferentially take up IsoLG-apoAI versus apoAI. Our data also show that IsoLG-apoAI directly upregulates expression of ABCA1 and SRBI in tubular cells. These data suggest that intrarenal accumulation of IsoLG-apoAI, reflects at least in part the avid uptake of filtered IsoLG-apoAI.

Our studies provide insights into renal handling of lipoproteins beyond the proximal tubule. Previous studies have substantiated a key role for kidneys in filtration and salvage of apoAI/HDL in maintaining plasma apoAI/HDL (19). Further, although lymphatics are a primary conduit for transport of apoAI/HDL out of the peripheral interstitium, the role of the renal lymphatic network in transport of reabsorbed apoAI has not been examined to our knowledge. We demonstrate colocalization of apoAI with podoplanin-positive lymphatics and document elevated apoAI in renal lymph of proteinuric versus uninjured animals. In parallel, IsoLG levels were increased in the renal lymph as well as in the renal lymph nodes of proteinuric versus CTL rats. The IsoLG modified apoAI affected the vascular dynamics of renal-collecting vessel lymphatics, increasing frequency, but reducing EDD, ESD, and amplitude of contraction compared with unmodified apoAI (Figure 9). These results comport with our recent study showing that PAN kidney injury diminishes lymphatic vessel pumping efficiency including reduced amplitude of contraction, which predicts reduced clearance of the renal interstitium (36). Indeed, ^23^Na-MRI revealed significant accumulation of sodium and water in the injured kidneys compared with normal CTLs.

Previous studies showed that oxidative modification of lipoproteins enhances cellular internalization that induces inflammation, immune cell activation, and cellular toxicity (23, 37, 38). Our data indicate that IsoLG-apoAI directly damages TECs and LECs. IsoLG-apoAI increased markers of cell injury, e.g., KIM-1 and inflammation, e.g., NLRP3, IL-1, and IL-6. Thus, in addition to proximal tubule cell damage by harmful albumin-associated cargo, urinary apoAI modified by IsoLG may be an additional cytotoxic mechanism. IsoLG-apoAI also increased VEGF-C in cultured TECs. In vivo, proteinuric mice had greater immunostaining of VEGF-C in TECs, and proteinuric rats showed twice as much VEGF-C in renal lymph as CTL animals. Both proteinuric mice and rats had significantly increased lymphatic vessel density that may reflect IsoLG-apoAI stimulation of VEGF-C production by proximal tubules. IsoLG-apoAI directly affected the phenotype of LECs, including increasing migration and proliferation and activation of inflammatory pathways (IL-1, IL-6, NLRP3, SPHK2, and SPNS2). IsoLG-apoAI constricted the lymphatic vessels, which could compromise interstitial clearance. Indeed, IsoLG-apoAI was cleared more slowly than the unmodified apoAI by normal kidneys (Figure 6). The slowed renal clearance of IsoLG-apoAI was further accentuated in proteinuric kidneys compared with uninjured kidneys. Collectively, our data suggest that proteinuric kidney injury increases filtration of IsoLG-modified apoAI that enters the renal parenchyma, where renal lymphatics transport the filtered/reabsorbed lipoproteins.

Our in vivo experiments demonstrate a key role for IsoLG in kidney injury. To our knowledge, the studies are the first to examine the effects of dicarbonyl scavenging in kidney injury and the first to demonstrate renal protection from in vivo treatment with a dicarbonyl scavenger, PPM. PPM significantly reduced urinary IsoLG but did not affect urinary excretion of a marker of systemic lipid peroxidation, namely, F2-isoprostane. This is notable, as urinary F2-isoprostane is reduced by treatment with the nonspecific antioxidant alpha tocopherol (39, 40). These data support the idea that the beneficial effects of PPM are not due to general inhibition of lipid peroxidation or metal ion chelation but to reactive lipid dicarbonyl scavenging. Indeed, PPM reduced albuminuria, KIM-1, interstitial fibrosis, and lymphangiogenesis. In vitro, PPM decreased KIM-1 and NLRP3 in TECs exposed to IsoLG-apoAI. In LECs, PPM decreased migration and inflammation. Thus, renoprotective mechanisms of PPM, at least in part, involve preventing formation of dicarbonyl adducts of apoAI, thereby preserving its anticytotoxic, anti-inflammatory effects in proximal TECs and LECs. PPM is expected to benefit injured tissues and organs producing or accumulating excess IsoLG. The protective effects of orally administered PPM on proximal tubules and kidney lymphatics reflect scavenging occurring systemically and/or within the kidneys. These findings complement recent reports that treatment with scavengers of dicarbonyl species is effective in conditions with elevated IsoLG, including atherosclerosis, hypertension, and inflammation-mediated carcinogenesis in the gastrointestinal tract (11, 23, 38, 41, 42).

In sum, the potentially novel IsoLG/apoAI/proximal tubule/lymphatic vessel pathway carries significant pathophysiological implications in kidney disease. This pathway implies that filtered proteins other than albumin, namely apoAI, may serve as an important supplier of potentially harmful molecules, namely IsoLG, into the tubulointerstitium. The pathway to convey lipoproteins out of the interstitial compartment involves the renal lymphatic vascular system. IsoLG-modified apoAI activates both TECs and LECs and alters lymphatic dynamics in a direction that would encourage renal interstitial stagnation. Given the detrimental effects of IsoLG-modified apoAI in tubules and lymphatics, the beneficial effects of dicarbonyl scavengers may provide a potentially novel therapeutic target for progressive kidney damage.

## Methods

### Animals.

The studies used Nphs1-hCD25 transgenic (NEP25, C57BL/6 background) mice expressing human CD25 on podocytes. The podocytes in this model can be selectively injured by injection of recombinant immunotoxin, anti-Tac (Fv)-PE38 (LMB2), resulting in albuminuria (24, 43). Adult male NEP25 mice (12 weeks old) were injected with LMB2 (1 ng/g BW, i.v., provided by Ira Pastan, Laboratory of Molecular Biology, National Cancer Institute, NIH, Bethesda, Maryland, USA) and compared with WT mice. Half of NEP25 and WT mice were treated with the dicarbonyl scavenger PPM (1 g/L) in drinking water starting at time of LMB2 injection and continuing until sacrifice. Two weeks after LMB2, urine was collected, mice were sacrificed, and kidneys were harvested.

Twelve-week-old male DT^+^ mice (C57BL/6 background), expressing γ-glutamyl transferase 1 (γGT1) DTR on proximal TECs, develop proximal tubular injury following injection of human DT (100 ng/kg BW, i.p.; Sigma). Urine was collected 2 weeks after injection. For distal tubular injury, 12-week-old male C57BL/6 mice received FA (240 mg/g, i.p.; Sigma) or vehicle. Urine was collected 2 weeks after injection.

Adult male Sprague-Dawley rats (200–225 g; Charles River) were fed normal rat chow (LabDiet) and water ad libitum. Proteinuric kidney injury was induced by a single i.p. injection of PAN (125 mg/kg BW; Sigma) while saline-injected rats served as CTLs (25). Eight days after injection, renal lymph was collected in a subset of anesthetized rats by cannulating the lymph duct with a glass pipette. Urine was collected, and kidneys and renal lymphatic nodes were harvested after sacrifice.

Mice and rats were housed under normal conditions, with a 12-hour light/12-hour dark cycle and free access to normal rodent chow and water. All animal procedures were approved by the IACUC at Vanderbilt University.

### Human participants.

A total of 50 patients, age 3–18 years, seeking care in the Vanderbilt Pediatric Nephrology Clinic were enrolled, as previously reported (18). We quantitated IsoLG levels in patients who had enough urine to perform the measurement and arbitrarily chose patients representing the main diagnostic categories: glomerulonephritis, nephrotic syndrome, transplantation, congenital anomalies of the kidney and urinary tract, and primary tubulopathies. Nineteen healthy CTLs were recruited from a general pediatric setting. Exclusion criteria for CTLs included history of kidney disease or hypertension, acute illness, or medications other than vitamins, contraceptives, antihistamines, inhaled steroids, or bronchodilators. The study was approved by the IRB at Vanderbilt University Medical Center (VUMC). A waiver of consent was granted for patients who submitted urine as part of their routine care. CTLs were consented and asked to provide a urine sample for study purposes. Clinical characteristics of these groups are shown in Supplemental Table 1.

### Composition of urine and renal lymph.

Albuminuria was measured as urine ACR using Albumin kit (Exocell) and QuantiChrom Creatinine Assay Kit (BioAssay Systems), respectively. ApoAI levels in urine and renal lymph (human: R&D Systems; and rat & mouse: MyBioSource), urine KIM-1 and NGAL (R&D Systems), and VEGF-C levels in renal lymph (MyBioSource) were measured by ELISA according to the manufacturer’s instructions. Total IsoLG-protein adducts were measured by LC/MS as IsoLG-Lys after complete proteolytic digestion of urine, renal lymph, and renal lymphatic node samples (44).

### IP of the apoAI fraction in human urine.

Samples with more than 10 mL of urine from patients and CTLs were concentrated (Thermo Fisher Scientific) and the concentrated urine was incubated with anti-apoAI Ab-biotin (Abcam). ApoAI-positive fractions were isolated using the EasySep Release Human Biotin Positive Selection Kit (Stemcell Technologies) following the manufacturer’s instructions.

### Renal handling of apoAI and IsoLG-apoAI.

While apoAI exists primarily in HDL particles, it readily dissociates from HDL. We used apoAI to perform this experiment since the small size of apoAI (28 kD) may provide a more precise picture of its filtration and reabsorption processes than the much larger HDL particle. ApoAI was labeled by Alexa Fluor 555 (Thermo Fisher Scientific) and (5/6)-TAMRA-SE (G-Biosciences) and was detected as red color with a fluorescence microscope. IsoLG-apoAI was labeled by Alexa Fluor 488 (Thermo Fisher Scientific) and FITC (G-Biosciences), and then exposed to 1 nmol IsoLG per 10 μg apoAI (2.8 molar equivalent IsoLG) and incubated for 4 hours at 37°C. IsoLG-apoAI was detected as green color with a fluorescence microscope (45). NEP25 and WT mice were simultaneously injected i.v. with 10 μg/g BW each of labeled apoAI and IsoLG-apoAI. The kidneys were harvested at 30 minutes and 180 minutes after injection. Kidney sections were fixed in 4% paraformaldehyde/PBS, dehydrated, and paraffin-embedded, and 2 μm sections were cut for assessment under a fluorescence microscope. Frozen tissues were homogenized and measured by spectroscopy. The results were calculated from a standard curve of labeled apoAI or IsoLG-apoAI.

### Histological assessments of kidney tissue.

Kidney sections were fixed in 4% paraformaldehyde/PBS, then paraffin-embedded, and 2 μm were sections cut for staining. For apoAI, podoplanin, VEGF-C, and LYVE-1 sections were microwaved (750 W, 5 minutes, 3 times) in citrate buffer (pH 6.0). For collagen I, 0.8% collagenase (Sigma) for 1 hour at 37°C was employed for antigen retrieval. Endogenous peroxidase was quenched by 3% hydrogen peroxide. Primary Abs were incubated overnight at 4°C by adding goat anti-mouse apoAI (1:200; Abcam), mouse anti-rat podoplanin Ab (1:1,000; Novus Biologicals), hamster anti-mouse podoplanin Ab (1:2,000; Thermo Fisher Scientific), mouse anti-mouse VEGF-C Ab (1:200; Abcam), rabbit anti-mouse LYVE-1 (1:50; Abcam), and rabbit anti-mouse collagen I (1:500 dilution; Abcam). The ImmPRESS HRP Detection kit (Vector Laboratories) was used for the secondary Ab according to primary Ab for 30 minutes. DAB was used to develop the chromogen. ApoAI, podoplanin, LYVE-1, VEGF-C, and collagen I–stained slides were photographed with AxioCam MRc5 (Carl Zeiss) under the same conditions. Podoplanin or LYVE-1–positive vessels per area were counted. The collagen I–positive area was analyzed using ImageJ software (NIH). All sections were examined without knowledge of the treatment protocol. Slides treated with nonspecific antisera instead of primary Ab were used as negative CTLs.

For ABCA1, SRBI, and ABCG1, sections were microwaved (750 W, 5 minutes, 3 times) in citrate buffer (pH 6.0). Endogenous peroxidase was quenched by 3% hydrogen peroxide. Sections with primary Abs were incubated overnight at 4°C by adding rabbit anti-mouse ABCA1 Ab (1:200; Novus Biologicals), rabbit anti-mouse SRBI Ab (1:200; Novus Biologicals), and rabbit anti-mouse ABCG1 Ab (1:200; Novus Biologicals). ImmPRESS reagent (Vector Laboratories) and Alexa Fluor 488 Tyamide SuperBoost (Invitrogen) were used as secondary Abs. The intensity of tubular ABCA1 and SRBI staining was semiquantitatively scored (on a scale of 0, trace 0.5, 1–3^+^) and presented as the average of 20 fields (original magnification, 20×). Double staining for megalin and apoAI was done using EDTA (pH 8.0) for antigen retrieval, followed by primary Ab mouse anti-mouse megalin (1:500; Novus Biologicals) at 4°C overnight. The secondary Abs used ImmPRESS reagent and Alexa Fluor 488 Tyamide SuperBoost. Sections were treated with citrate buffer for antigen retrieval and incubated with goat anti-mouse apoAI at 4°C overnight, followed by anti-goat HRP (ImmPRESS reagent) and Alexa Fluor 546 Tyamide SuperBoost. Double staining for apoAI and podoplanin was done using citrate buffer for antigen retrieval followed by primary Ab apoAI at 4°C overnight. ImmPRESS reagent (Vector Laboratories) and Alexa Fluor 488 Tyamide SuperBoost were used as secondary Abs. Sections were then incubated with hamster anti-mouse podoplanin Ab at 4°C overnight, followed by biotinylated anti-hamster Ab (Vector Laboratories), ABC reagent, and Alexa Fluor 546 Tyamide SuperBoost (Invitrogen).

### Measurement of lymphatic dynamics ex vivo.

Rat renal lymphatic vessels were collected and mounted in a perfusion chamber for pressure myography assays (46, 47). Chambers were placed on inverted microscopes equipped with a digital image capture system (IonOptix) to record prevalve intraluminal diameters and frequency of contractions. Vessels were warmed to 37°C, pressurized to 0.5 mmHg using a column of Krebs buffer, and allowed to equilibrate (20–60 minutes). Vessels that failed to contract spontaneously were excluded from further study. Viable vessels were then pressurized in a stepwise manner to a constant pressure of 3.5 mmHg, then exposed to purified apoAI or modified apoAI using 1 molar equivalent synthetic IsoLG or vehicle (DMSO) (23). Fresh Krebs buffer was circulated to facilitate wash out.

### Cell culture.

Primary human renal TECs (RPTEC; Lonza) were cultured with conditioned renal epithelial cell growth medium (Lonza). Primary adult dermal LECs (HMVEC-DLyAd; Lonza) were cultured with conditioned endothelial growth medium (Lonza). Cells at passage 5–6 with approximately 70% confluence were starved in serum-free medium overnight and then incubated with unmodified or IsoLG-modified apoAI (apoAI: 10 μg/mL; and IsoLG: 1 μM/L) ± PPM (10 μM/L) for 24 hours (for TECs) or 18 hours (for LECs). Previously, we showed that this concentration of IsoLG yields levels of IsoLG-Lys adducts observed in vivo and does not produce unreacted IsoLG (23). Supernatants and cells were collected. VEGF-C in the cell lysates of cultured TECs was measured by Quantikine ELISA kit (R&D Systems).

To knock down ABCA1 and SRBI genes in TECs, we used Lipofectamine RNAiMAX Transfection system. Cells were transfected with ABCA1 and SRBI or nontargeting CTL siRNA (Dharmacon) at 100 nM final concentration (diluted with Opti-MEM) and incubated for 48 hours at 37°C. Transfection efficiency of TECs is described in the results (Figure 4, C and D).

### Cell proliferation, migration, and uptake of apoAI.

LECs proliferation assay was performed using the XTT cell viability kit (Cell Signaling Technology). The relative migratory ability of LECs was determined by wound-healing assay. Cells were plated onto 6-well plates and allowed to reach full confluence before a scratch was gently created with a pipette tip. The area devoid of cells was photographed at the time the scratch was created and at 23 hours. The relative distance of LECs migrated into the denuded area was measured.

To assess apoAI uptake by TECs in vitro, we used apoAI labeled with Alexa Fluor 555 and (5/6)-TAMRA-SE, which was exposed to IsoLG and incubated for 4 hours at 37°C. TECs with approximately 70% confluence were starved in serum-free medium overnight then incubated with fluorescence labeled unmodified or IsoLG-modified apoAI ± PPM. After 4 hours, the cell lysates were collected for fluorescent measurements (48). In complementary studies, TECs grown on slides were costained with actin (Invitrogen) and mounting solution containing DAPI.

### Western blot.

Total protein of TECs was extracted by RIPA buffer containing EDTA, EGTA, phosphatase inhibitor, and protease inhibitor (Roche). Equal amounts of total proteins were separated by NuPAGE 4%–12% Bis-Tris gel electrophoresis and electrophoretically transferred to nitrocellulose membranes by iBlot system (Invitrogen) blocked with 5% nonfat milk in Tris-buffered saline containing 0.1% Tween 20 (TBS-T) and incubated with primary Ab for ABCA1 (1:3,000), SRBI (1:3,000), or β-actin (1:20,000; Sigma) at 4°C overnight. After washing, HRP-labeled at IgG secondary Abs (1:3,000 for rabbit in 5% powdered nonfat milk/TBS-T; Promega) were added and incubated at room temperature for 1 hour. Protein bands were visualized by Western Lightning Plus-ECL (PerkinElmer). Abundance of protein expression shown as a specific band was analyzed by ImageJ software, normalized by loading CTL (β-actin).

### Quantitative real-time PCR.

Total RNA was isolated from kidney cortex lysates, TECs, and LECs by RNase Mini Kit (QIAGEN). Reverse transcription was performed using the High-Capacity cDNA Reverse Transcription Kit (Applied Biosystems). Quantitative real-time PCR was performed in a total reaction volume of 25 μL using 12.5 μL Universal Master Mix II, 1.25 μL forward and reverse primers KIM-1 (*HAVCR1*), *VEGFC*, *SPHK2*, *SPNS2*, *IL-1*, *IL-6*, and *NLRP3* (Thermo Fisher Scientific), and 11.25 μL cDNA (10 ng/μL). Quantitative real-time PCR was carried out using the CFX96 Real-Time PCR Detection System (Bio-Rad Laboratories) with the following cycling parameters: polymerase activation for 10 minutes at 95°C and amplification for 40 cycles of 15 seconds at 95°C and 60 seconds at 60°C. Experimental Ct values were normalized to β-actin measured on the same plate, and fold differences in gene expression were determined using the 2^–ΔΔCt^ method (49).

### Statistics.

Results are expressed as means ± SEM. Statistical significance was determined by Wilcoxon rank sum test. For more than 2 groups, statistical significance was determined by Kruskal-Wallis test followed by Wilcoxon rank sum tests and Bonferroni correction. *P* < 0.05 was considered significant.

### Study approval.

All procedures were approved by the IACUC of VUMC and conducted according to the NIH’s *Guide for the Care and Use of Laboratory Animals* (National Academies Press, 2011). Collection of human urine was approved by the Institutional Review Board at VUMC. A waiver of consent was granted for patients who submitted urine as part of their routine care.

## Author contributions

JZ performed experiments in vivo and in vitro and helped in the writing and editing of the manuscript. HCY collected renal lymph, performed histologic scoring, and helped with the experimental design and editing of the manuscript. ELS performed the ex vivo lymphatic dynamic experiments and editing of the manuscript. TM provided the NEP25/megalin KO as well as NEP25/megalin mosaic mice. AJC collected the human urine samples. VY measured IsoLG protein adducts. ZM measured IsoLG-protein adducts. LSMZ performed IsoLG modifications of apoAI and measured resulting protein adducts. MFL provided funding and edited the manuscript. ABF provided the DT- and FA-treated mice and helped in the writing and editing of the manuscript. AK helped in data analysis and edited the manuscript. SSD supervised the measurements of IsoLG protein adducts and IsoLG modifications of apoAI, analyzed data, and edited the manuscript. VK conceived of the project, oversaw the experiments, and wrote and edited the manuscript.

## Supplementary Material

Supplemental data

## Version 1 09/20/2022

In-Press Preview

## Version 2 11/08/2022

Electronic publication
