# Supplementary material for: Dicarbonyl-modified lipoproteins contribute to proteinuric kidney injury
Source: JCI Insight. 2022 Nov 8;7(21):e161878. doi: 10.1172/jci.insight.161878 (PMC9675465; doi:10.1172/jci.insight.161878)
Supplement: Supplemental data [file jciinsight-7-161878-s228.pdf]

Supplemental Table 1: Demographic and clinical characteristics of patients and controls

	Controls (n=19)	Patients (n=50)
Sex		
Female	53% (10)	38% (19)
Male	47% (9)	62% (31)
Age (yrs)	15.32±0.89 (19)	11.56±0.74 (50)
Race*		
White	37% (7)	62% (31)
Black	0% (0)	14% (7)
Asian	16% (3)	4% (2)
Primary diagnosis		
Glomerulonephritis		18% (9)
Nephrotic syndrome		30% (15)
Transplant		22% (11)
Dysplasia/CAKUT		22% (11)
Others		8% (4)
GFR Schwartz (ml/min/1.73m^2) **	83.60±4.84 (5)	62.71±4.83 (38)
Urine albumin/creatinine (ACR) (mg/g)	11.57±2.50 (13)	217.00±48.42 (45)
Urine apoAI/creatinine (µg/mg)	0.21±0.15 (16)	2.89±1.12 (45)

x±s:mean±SE, n in parentheses

*Race extracted from charts

**GFR documented within 6 months of the study visit

Supplemental Table 2: Summary of results in patients and animal models

Model	Species	Nephron segment affected	ACR	Urinary apoAI	Urinary IsolG
Varying disease	Human	Varied	↑ ↑ ↑	↑ ↑ ↑	↑
NEP25	Mouse	Glomerulus	↑ ↑ ↑	↑ ↑ ↑	↑
PAN	Rat	Glomerulus	↑ ↑ ↑	↑ ↑ ↑	↑
DT	Mouse	Proximal tubule	↑ ↑	↑	→
FA	Mouse	Distal tubule	↑	→	NA

↑ = increased, → = no change, NA = not available

NEP25: Nphs1-hCD25 transgenic mice; PAN: Puromycin aminonucleoside; DT: Diphtheria toxin; FA: Folic acid; ACR: Urinary albumin:creatinine ratio; apoAI: Urinary apolipoprotein AI; IsoLG: Isolevuglandin
